# The Relationship Between Range of Motion and Injuries in Adolescent Dancers and Sportspersons: A Systematic Review

**DOI:** 10.3389/fpsyg.2018.00287

**Published:** 2018-03-22

**Authors:** Joyce M. Storm, Roger Wolman, Eric W. P. Bakker, Matthew A. Wyon

**Affiliations:** ^1^Research Centre for Sport, Exercise and Performance, Institute for Sport and Human Sciences, University of Wolverhampton, Walsall, United Kingdom; ^2^National Institute of Dance Medicine and Science, Birmingham, United Kingdom; ^3^Department of Rheumatology and Sport and Exercise Medicine, Royal National Orthopaedic Hospital, Stanmore, United Kingdom; ^4^Division of Clinical Methods and Public Health, Academic Medical Centre, University of Amsterdam, Amsterdam, Netherlands

**Keywords:** flexibility, injury incidence, ballet, adolescent, puberty, delayed

## Abstract

**Background:** The frequent and intensive training and performance of pre-professional ballet dancers and sportspersons is offered at a time when young ballet dancers and young athletes may be vulnerable to injury due to the progress through adolescence and growth spurts.

**Hypothesis:** There are changes in range of motion during the progress through adolescence and growth periods in dancers and sportspersons. These changes in ROM can be linked to the increase of injury.

**Objectives:** The primary aim of this systematic review is to determine whether there are changes in ROM during the progress through adolescence and growth spurts in dancers and sportspersons. The secondary aim is to determine whether these changes can predict the risk of injuries for adolescent dancers and sportspersons.

**Search strategy:** Pubmed, Cochrane Register of Controlled Trails (CENTRAL), Cochrane Database of Systematic Reviews (CDSR), EBSCO Host databases: CINAHL Plus, MEDLINE, SPORTDiscus, Embase were searched using MeSH terms. Manual search in the Journal of Dance Medicine and Science and screening of the reference lists of identified studies and reviews was conducted.

**Selection criteria:** Studies included adolescent dancers and sportspersons, aged 8–18, both sexes, growth spurt related to changes in ROM and injury incidence.

**Data collection and analysis:** Search strategy was performed in the flow diagram of the Preferred Reporting Items for Systematic Reviews and Meta-Analyses (PRISMA). Two reviewers independently appraised each included study using Strengthening the Reporting of Observational Studies in Epidemiology (STROBE) for methodological quality of the included studies. For data extraction, the following information was systematically extracted: first author and year of publication, study design, participants (sample size of mean age), age, maturation (if assessed), intervention, outcome(s), and some notes of each study. For evaluation of the risk of bias and precision the Research Triangle Institute Item Bank (RTI-IB) is included.

**Main results:** Seven observational studies met the inclusion criteria of this current review. The results of this review suggest that there are changes in ROM during the progress through adolescence and growth spurts in dancers and sportspersons. These changes may lead to an increase in injury incidence.

**Conclusion:** There is evidence linking to changes in ROM during the progress through adolescence and growth spurts in dancers and sportspersons. These changes in ROM may be related to injury incidence.

## Introduction

For those wanting to have careers in sport and dance, it has been established that dedicated training (high volume and intensity) starts at a relatively young age (Lloyd and Oliver, 2012). Both dancers (Hamilton et al., 1992; Koutedakis and Jamurtas, 2004) and sportspersons are exposed to extreme physical demands on the human body (Liederbach, 1985; Twitchett et al., 2009; Gil et al., 2013; Smith et al., 2015) and therefore are subject to increased injury risk (Price et al., 2004; Allen et al., 2012, 2013). Young promising sports people and dancers are committed to full-time pre-professional training by the age of 11. Pre-professional ballet training places increasing demands on the student and is designed to replicate both the technical demands and intensity of the professional setting, whilst sport development is often within academy settings (Premier League, 2011). For these adolescent students, it often requires up to 5 or 6 days per week of training as they approach the professional level (Ekegren et al., 2014; Caine et al., 2015). The frequent and intensive training and performance of pre-professional ballet dancers and sportspersons is offered at a time when young ballet dancers and young athletes are progressing through adolescence and growth spurts. This can affect the maturing growth plates and the growth process itself (Poggini et al., 1999; Caine et al., 2016). The growth spurt can increase the risk of underdeveloped motor skills and spatial perception due to altered biofeedback (Caine et al., 2015). Compared to young sportspersons, dancers require an extreme Range of Motion (ROM) and fine motor control to achieve the technical demands of ballet, such as repetitive and rotational movements and point work, in a professional setting (Poggini et al., 1999). This may expose these young dancers and sportspeople to a higher risk of injuries (Caine et al., 2015).

### Risk of injuries

Compared to most athletic activities, research on injury incidence and risk factors in dance is limited (Ekegren et al., 2014). Therefore, young adolescence sportspersons are also included in this current review. This offers a broader generalizability of this review to a wider exercising population.

For dancers in pre-professional training, usually aged between 11 and 18 years old in ballet but can be up to 22 years old in other genres, two studies reported incidence and risk factors (Luke et al., 2002; Gamboa et al., 2008). The incidence of dance injury in these studies has been reported to be between 0.62 and 5.6 injuries per 1,000 dancing h (Luke et al., 2002; Gamboa et al., 2008). Another study reported a clinical incidence of injury of 1.42 injuries per dancer and the risk of injury 76% over a one-year period in elite pre-professional ballet students, aged 15–19 years (Ekegren et al., 2014). The rate of injury in this study was 1.38/1,000 h of dance and 1.87/1,000 dance exposures. This study (Ekegren et al., 2014) also showed that compared to young adolescent sportspersons pre-professional dancers had a higher risk of injuries, which may be attributable to the intensive and high level of ballet training. Adolescent dancers reported a considerably lower injury incidence than professional dancers; adolescent dancers: 32–51% vs. professional 67–95% (Gamboa et al., 2008). Smith et al. (2015) reported overall injury incidence among amateur (mean age 16 years) and professional (mean age 27 years) dancers was calculated to be 0.97 and 1.24 injuries per 1,000 dance h; in both genders female dancers had a higher injury incidence: male (0.99 vs. 1.06) and female (1.09 vs. 1.46). However, this study only reported overuse injuries and despite of this, the injury incidence in this study is low.

In case of professional dancers, one study (Allen et al., 2012) showed an overall injury incidence of 4.4 injuries per 1,000 h (female, 4.1; male, 4.8; *P* > 0.05) and a mean of 6.8 injuries per dancer (female, 6.3; male, 7.3; *P* > 0.05). In this study most injuries were counted as overuse injuries (64%; female, 68%; male, 60%; *P* > 0.05) and traumatic injuries (32% for females; 40% for males; *P* < 0.05). Finally, a systematic review (Allen et al., 2014) reported a mean injury incidence of 1.33 per 1,000 h of dance, with one study deceasing injury incidence from 2.46/1,000 to 0.84/1,000 h of dance in professional dancers by implementing a comprehensive medical management.

### Intrinsic and extrinsic factors

To investigate the association between range of motion and the risk of injury and changes in performance, the influence of intrinsic as well as extrinsic factors need to be considered. Pre-professional training is offered at a time when young ballet dancers and sportspersons may be vulnerable to injury due to the presence of maturing growth plates and the growth process (intrinsic factors) itself (Poggini et al., 1999; Caine et al., 2016). These intrinsic factors may place the young adolescence dancers and sportspersons at a higher risk of injury (Caine et al., 2015). Some studies suggest that intrinsic factors, such as a temporary decrease of proprioception during adolescence, strength, and flexibility imbalances and poor postural alignment have a causal relationship with dance injuries (Gamboa et al., 2008). Unfortunately, very few studies have managed to investigate the relationships between measurement of intrinsic characteristics and dance injuries (Gamboa et al., 2008). On the other hand extrinsic factors, such as the kind of sports activities, the level of performance cannot be overlooked and need to be considered. They may also have an influence on injury (Coplan, 2002; Negus et al., 2005). For example, one study suggested that the range of motion of hip rotation decreases because of playing soccer (de Castro et al., 2013).

### Range of motion (ROM)

To determine the association between range of motion and the risk of injury and changes in performance, it is important to understand why range of motion might change and if these changes in ROM can be linked to injury and performance. Recently, a study showed that stretching within a warming-up can reduce muscle injuries and can increase the ROM of the joints, which may influence the athletic performance (Behm et al., 2016). For a better understanding of the association between range of motion and the risk of injury, the ROM itself also need to be considered as it is determined by a number of anatomical, biomechanical and physiological factors such as bone shape, connective tissue, muscle mass and neurological tissue (Baechle and Earle, 2008).

However, there is still little known about the ROM in dancers and sportspersons as compared with non-dancers of the same age and the changes in ROM in relation to growth and development (Steinberg et al., 2006). Therefore, the hypothesis of this current review is: “There are changes in range of motion during the progress through adolescence and growth periods in dancers and sportspersons. These changes in ROM can be linked to the increase of injury.” First, it will be researched if there are changes in ROM during adolescence in dancers and sportspersons. Second, if there are changes exist, will these changes have a causal relationship with injury incidence. Therefore, the research question of this present review is: “Is there a relationship between changes in range of motion (ROM) during adolescent growth spurts in dancers and sportspersons and do these changes affect injury incidence?”

Furthermore, to investigate the research question the primary aim of this systematic review is to determine whether there are changes in range of motion during the progress through adolescence and growth spurts in dancers and sportspersons. The secondary aim is to determine whether these changes can predict the risk of injuries for adolescent dancers and sportspersons.

## Method

### Search strategy

**Figure 1:**
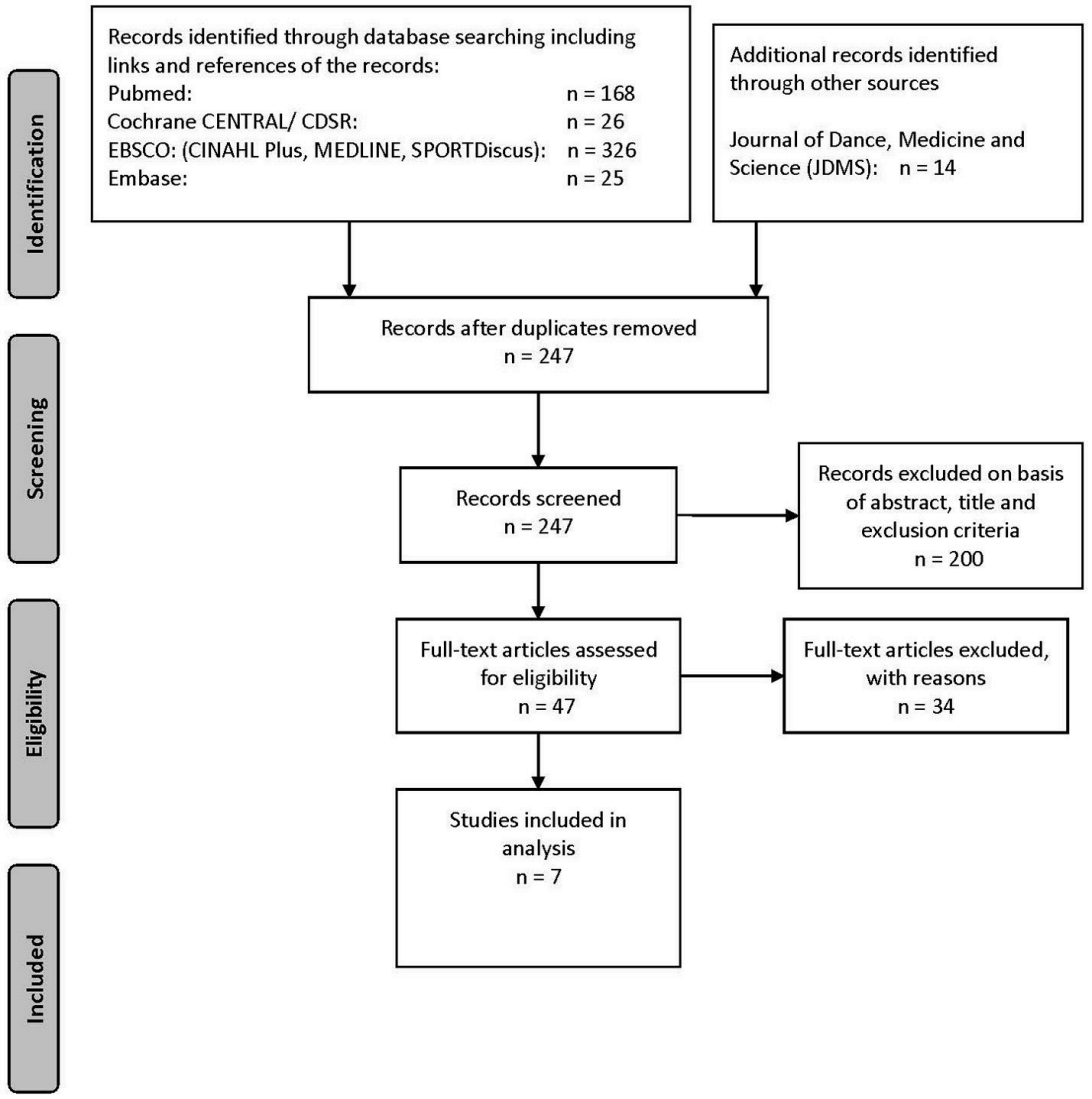
Prisma flow diagram.

Using the Preferred Reporting Items for Systematic Reviews and Meta-Analyses (PRISMA) guidelines (Figure 1; Moher et al., 2009, 2015; Welch et al., 2016), the following databases were searched using MeSH terms, free text, keywords and subheadings relating to the research question of this present review (Appendix 1 in Supplementary Material).

1. Pubmed,
2. Cochrane Register of Controlled Trails (CENTRAL),
3. Cochrane Database of Systematic Reviews (CDSR),
4. EBSCO Host databases: CINAHL Plus, MEDLINE, SPORTDiscus, and Embase.

In addition, a manual search in the Journal of Dance Medicine and Science was performed for publications from March 1997 till March 2017 and screening of the reference lists of identified studies and reviews was conducted.

### Criteria for considering studies for this review (in-/exclusion)

There were no restrictions for the type of studies and none for languages. Articles still in progress or not published yet were excluded. Adolescent pre-professional dancers and sportspersons participants aged 8–18 years of either gender were included. Adult dancers, professional dancers and sportspersons were excluded. For the outcome of this review, the focus was on growth spurt related to changes in ROM, injury incidence.

### Study selection

One reviewer (JMS) screened titles and abstracts of all studies identified by the search strategy. Potentially relevant studies were selected from each database and imported in EndNote X6. In EndNote X6 duplicates were removed. Forty-seven selected full-text articles were retrieved and reviewed. Consensus between two reviewers (MW and JMS) was used to resolve disagreements concerning the final inclusion of one study. Finally, 34 publications that did not meet the criteria (Table 1) were excluded. Seven studies met the inclusion criteria of this review (Figure 1).

**Table 1:** Characteristics of excludes studies.

**Study**	**Reasons for exclusion**
1. Bayraktar, B. l. (2011). “Frequent injuries in adolescent athletes.” Turkish Pediatrics Archive/Turk Pediatri Arsivi 46, 43-45.	No high methodological quality
2. Bennell, K., et al. (1999). “Hip and ankle range of motion and hip muscle strength in young female ballet dancers and controls.” Br J Sports Med 33(5): 340-346.	Not related to the inclusion criteria/objectives of this review (rom not related to growth spurt)
3. Bennell, K. L., et al. (2001). “Changes in hip and ankle range of motion and hip muscle strength in 8-11 year old novice female ballet dancers and controls: a 12 month follow up study.” Br J Sports Med 35(1): 54-59.	Not related to the inclusion criteria/objectives of this review (changes ROM not related to growth spurt)
4. Cameron, N. (2014). “Growth and development and athletic performance. / rast in razvoj ter uspešnost v športu.” Kinesiologia Slovenica 20(3): 5-13.	No high methodological quality, not related to the inclusion criteria of this review
5. De Castro, J. V., et al. (2013). “Incidence of Decreased Hip Range of Motion in Youth Soccer Players and Response to a Stretching Program: A Randomized Clinical Trial.” J Sport Rehabil 22(2): 100-107.	Not related to the inclusion criteria/objectives of this review
6. Georgopoulos, N. A., et al. (2001). “Height velocity and skeletal maturation in elite female rhythmic gymnasts.” The Journal Of Clinical Endocrinology And Metabolism 86(11): 5159-5164.	It's about the progression of growth in rhythmic gymnasts. No growth spurt related to changes in ROM or injury incidence but to a late acceleration of linear growth.
7. Gerrard, D. F. (1993). “Overuse injury and growing bones: the young athlete at risk.” Br J Sports Med 27(1): 14-18.	No high methodological quality
8. Kadel, N. J., et al. (2005). “Anthropometric measurements of young ballet dancers: examining body composition, puberty, flexibility, and joint range of motion in comparison with non-dancer controls.” Journal of Dance Medicine & Science 9(3/4): 84-90	Not related to the inclusion criteria/objectives of this review
9. Karahan, M. and B. Erol (2004). “[Muscle and tendon injuries in children and adolescents].” Acta Orthop Traumatol Turc 38 Suppl 1: 37-46.	Not related to the inclusion criteria/objectives of this review
10. Karlsson, M. K., et al. (2008). “Physical activity increases bone mass during growth.” Food & Nutrition Research 52: 1-10.	Not related to the inclusion criteria/objectives of this review
11. Khan, K., et al. (1997). “Hip and ankle range of motion in elite classical ballet dancers and controls.” Clin J Sport Med 7(3): 174-179.	Not related to the inclusion criteria/objectives of this review (ROM dancers compared to controls)
12. Khan, K. M., et al. (2000). “Can 16-18-year-old elite ballet dancers improve their hip and ankle range of motion over a 12-month period?” Clin J Sport Med 10(2): 98-103.	Not related to the inclusion criteria/objectives of this review
13. Kibler, W. B. and T. J. Chandler (2003). “Range of motion in junior tennis players participating in an injury risk modification program.” J Sci Med Sport 6(1): 51-62.	Not related to the inclusion criteria/objectives of this review
14. Lantz, J. M. and A. Kavchak-Emerson (2014). “Lower extremity maturational variation in adolescent girls may predispose to sports-related knee injury.” J Pediatr 164(1): 216-219.	Commentary, no study
15. Malina, R., et al. (2013). “Role of Intensive Training in the Growth and Maturation of Artistic Gymnasts.” Sports Medicine 43(9): 783-802.	Not related to the inclusion criteria/objectives of this review. It's about the effects of training on growth etc.
16. Marius Meylan, C., et al. (2014). “Adjustment of Measures of Strength and Power in Youth Male Athletes Differing in Body Mass and Maturation.” Pediatric Exercise Science 26(1): 41-48.	No high methodological quality, not related to the inclusion criteria of this review
17. Matthews, B. L., et al. (2006). “Dancing for bone health: a 3-year longitudinal study of bone mineral accrual across puberty in female non-elite dancers and controls.” Osteoporosis International: A Journal Established As Result Of Cooperation Between The European Foundation For Osteoporosis And The National Osteoporosis Foundation Of The USA 17(7): 1043-1054.	Not related to the inclusion criteria/objectives of this review
18. Matthews, B. L., et al. (2006). “The influence of dance training on growth and maturation of young females: A mixed longitudinal study.” Annals of Human Biology 33(3): 342-356.	Not related to the inclusion criteria/objectives of this review It's about the effects of training on growth etc.
19. Michaud, P. A., et al. (2001). “Sports activities related to injuries? A survey among 9-19 year olds in Switzerland.” Injury Prevention: Journal Of The International Society For Child And Adolescent Injury Prevention 7(1): 41-45.	No high methodological quality, a survey
20. Myer, G. D., et al. (2009). “Longitudinal assessment of non-contact anterior cruciate ligament injury risk factors during maturation in a female athlete: a case report.” J Athl Train 44(1): 101-109.	No high methodological quality, a case report
21. O'Brien, T. D. (2016). “Musculoskeletal Proportionality, Biomechanical Considerations, and Their Contribution to Movement in Adults and Children.” Pediatric Exercise Science 28(2): 210-216.	No high methodological quality, not related to the inclusion criteria of this review. An article
22. Pfeiffer, R. P., et al. (2002). “Acute injuries to the lower extremities in pediatric and adolescent athletes.” Athletic Therapy Today 7(6): 18-72.	No high methodological quality, not related to the inclusion criteria of this review.
23. Philippaerts, R. M., et al. (2006). “The relationship between peak height velocity and physical performance in youth soccer players.” J Sports Sci 24(3): 221-230.	Not related to the inclusion criteria/objectives of this review (no changes in ROM)
24. Phillips, C. (1999). “Strength Training of Dancers during the Adolescent Growth Spurt.” Journal of Dance Medicine & Science 3(2): 66-72.	No high methodological quality, not related to the inclusion criteria of this review.
25. Pigeon P, et al: Intensive dance practice. Repercussions on growth and puberty. Am J Sports Med 25(2):243-247, 1997.	Not related to the inclusion criteria/objectives of this review
26. Quatman-Yates, C. C., et al. (2013). “A longitudinal evaluation of maturational effects on lower extremity strength in female adolescent athletes.” Pediatr Phys Ther 25(3): 271-276.	Not related to the inclusion criteria/objectives of this review
27. Roemmich, J. N. and A. D. Rogol (1995). “Physiology of growth and development: Its relationship to performance in the young athlete.” Clin Sports Med 14(3): 483-502.	Not related to the inclusion criteria/objectives of this review
28. Schmitz, R. J., et al. (2009). “Dynamic Valgus Alignment and Functional Strength in Males and Females During Maturation.” Journal of Athletic Training (National Athletic Trainers' Association) 44(1): 26-32.	Not related to the inclusion criteria/objectives of this review
29. Sigward, S. M., et al. (2012). “Influence of sex and maturation on knee mechanics during side-step cutting.” Med Sci Sports Exerc 44(8): 1497-1503.	Not related to the inclusion criteria/objectives of this review
30. Steinberg, N., et al. (2014). “Injuries among talented young dancers: findings from the U.K. Centres for Advanced Training.” Int J Sports Med 35(3): 238-244.	Not related to the inclusion criteria of this review (focus on the injuries and not so much on the relation with growth)
31. Steinberg, N., et al. (2016). “Joint Hypermobility and Joint Range of Motion in Young Dancers.” J Clin Rheumatol 22(4): 171-178.	Not related to the inclusion criteria/objectives of this review
32. Steinberg, N., et al. (2012). “Extrinsic and intrinsic risk factors associated with injuries in young dancers aged 8-16 years.” J Sports Sci 30(5): 485-495.	Not related to the inclusion criteria/objectives of this review
33. Tait, T. J., et al. (2016). “Does maturational timing influence the leg length, leg strength velocity relationships in adolescent boys?” Pediatric Exercise Science 28: 34-34.	Not related to the inclusion criteria/objectives of this review
34. Wild, C. Y., et al. (2016). “How Young Girls Change Their Landing Technique Throughout the Adolescent Growth Spurt.” Am J Sports Med 44(5): 1116-1123.	Not related to the inclusion criteria/objectives of this review

### Data extraction

The following information was systematically extracted by one reviewer (JMS): first author and year of publication, study design, participants (sample size of mean age), age, maturation (if assessed), intervention, outcome(s) and some notes of each study. After discussion between JMS and MW about the data extraction form, an agreement was reached and “the level of evidence” was added as a separate column (Table 2).

**Table 2:** Summary of included studies.

**Study: First author, year of publication**	**Design**	**Level of evidence**	**Participants, sample size of mean age**	**Age**	**Maturation (if assessed)**	**Intervention**	**Outcome(s)**	**Notes**
Ford et al., 2010	Mixed cross-sectional/longitudinal (cohort/cross-sectional)	2 (cohort) and 3 (cross-sectional)	Female; *n* = 265, male; *n* = 50 (basketball, soccer)	12.3–15.4 years (pubertal; *n* = 182: age 12.4 ± 0.9 years and post-pubertal; *n* = 133: age 14.5 ± 1.4 years)	Pubertal or post-pubertal (PMOS)	2 testing sessions, 1 year apart: active joint stiffness of the ankle, knee and hip was estimated during a drop vertical jump (DVJ).	Both pubertal male and female individuals longitudinally increased stiffness active knee. Males had active stiffness ankle and hip. In the post-pubertal group, male individuals had peak ankle and hip moments, females had a higher knee to hip moment.	Sample size groups are different. Pubertal: female; *n* = 145, male; *n* = 37 and the post-pubertal groups: female; *n* = 120 and male; *n* = 13. The generalizability of its findings is limited because only soccer and basketball players were included.
Hewett et al., 2004	Cross-sectional		Female; *n* = 100, male; *n* = 81 (high-school and middle school soccer and basketball players)	11.5–15.5 years	Pre-pubertal (TS I); pubertal (TS II–III) and post-pubertal (TS IV–V) (based on PMOS)	Test: Drop vertical jump (DVJ). Dynamic control of the knee joint was measured kinetically by assessing knee joint torques; the results of the testing sessions were compared between female and male athletes, depending on the maturational stage. The lower extremity bone length was measured with three-dimensional kinematic analysis.	No significant differences between male and female individuals in the pre-pubertal or pubertal groups. In the pubertal group, females showed an increased valgus angle at initial contact, peak valgus and increased medial knee motion compared to males. Females showed a higher significant knee valgus and medial knee motion with age, which was absent in male individuals. Male athletes demonstrated an increased quadriceps peak torque with increasing maturity, whereas females did not.	The number of participants in the female groups differs from the male groups throughout every maturation stage. Furthermore, there are several possible contributing and confounding variables that were not controlled: school, team, age/grade, aggressiveness, foot pronation, quadriceps angle, femoral notch width, and blood hormone levels. This study had a cross-sectional nature, not a longitudinal.
Hewett et al., 2015	Longitudinal/ cross-sectional controlled laboratory study		Female; *n* = 674, male; *n* = 218 (high-school and middle school soccer and basketball players)	10–18 years	Maturation was caught by estimates of percent (%) adult stature. Participants were defined mature as 92% of adult stature or greater.	DVJ	Mature females showed increased peak knee abduction moment (KAM) and knee abduction angles (KAA) related to growing adolescent females. KAM peaked in females at peak height velocity (PHV) and the KAM peak increases in both females and males after the arise of adolescence. After PHV the dynamic knee control of males started to increase again, whereas dynamic knee stability in females still decreases.	There are some possible contributing and confounding variables that were not controlled; school, team, age/grade, aggressiveness, foot pronation, quadriceps angle, femoral notch width, and blood hormone levels. The inclusion of only soccer and basketball players may be a limitation to the generalizability of the findings.
Quatman et al., 2006	Cohort; a prospective, controlled, longitudinal laboratory study	2	Female; *n* = 16, male; *n* = 17. Participants were evaluated for 2 consecutive years. Subjects are preadolescent and adolescent male and female athletes (middle and high school). Participants were included if they were classified as pubertal during the first year of testing and post pubertal during the second year.	12.6–14.8 years	Prepubertal (TS I), early pubertal (TS II-III), post-pubertal (TS IV-V) (based on PMOS)	DVJ: ground-reaction force and vertical jump height were measured.	The male athletes showed increased vertical jump height with maturation (*P* < 0.001); female athletes did not. Males significantly reduced their landing ground-reaction force (*P* = 0.005), females did not. Take off force decreased in females (*P* = 0.003), not in males. Both males and females had decreased loading rates with maturation (*P* < 0.001) but females had higher loading rates at both stages of maturation (*P* = 0.037) compared to males.	1 male athlete was excluded because of data collection error. The female and male groups were not height and weight matched.
Quatman et al., 2008	Cross-sectional/cohort		Female; *n* = 275, male; *n* = 143 (middle and high school basketball and soccer athletes)	11–18 years	Pre-pubertal (TS 1), pubertal (TS ll-lll), post-pubertal (TS IV-V)	Generalized joint laxity assessed using the Beighton and Horan Joint Mobility Index (BHJMI).	Females showed increased generalized joint laxity scores between pre-pubertal and post-pubertal groups (*P* = 0.042), while males did not (*P* = 0.582). Gender differences in BHJMI score was found at puberty and post-puberty (P < 0.001).	TS (Tanner Staging) was not used. The reliability and reproducibility of the generalized joint laxity testing can be discussed. Another limitation can be the cross-sectional nature; changes over time within the participants cannot be captured.
Steinberg et al., 2006	Cross-sectional	3	Female; *n* = 1320 (classical ballet, modern dance, jazz, etc) and a control group of non-dancers; *n* = 226	8–16 years		Range of motion was measured for the hip, knee, ankle, foot and spinal joints.	Combined ankle and foot plantar flexion (pointe), ankle plantar flexion and hip external rotation showed no change in range of motion in dancers. Range of motion diminished with age in the non-dancers. For ankle dorsiflexion, neither group showed any change with age and range of motion was significantly more increased in the non-dancer group. The range of motion decreased with age in both groups for knee flexion, hip flexion, and hip internal rotation. The ROM of hip abduction decreased with age in dancers and did not change in the non-dancers. For hip extension the range of motion increased in both groups. The range of motion of the lower back and hamstrings increased among dancers with age and remained constant among non-dancers.	There is a large sample of dancers compared to a small control group and the cross-sectional nature can be a limitation.
Wild et al., 2013	Longitudinal	3	Female; *n* = 33	10–13 years	TS II and 4–6 months from the PHV	Participants were tested up to four times during the 12 months of their growth spurt, according to the timing of their maturity offset (test 1: maturity offset = −6 to −4 months; test 2: maturity offset = 0 months; test 3: maturity offset = +4 months; test 4: maturity offset = +8 months). During each testing session, anterior knee laxity, lower limb flexibility and isokinetic strength as well as saliva measures of estradiol concentration were measured.	A significant (*P* = 0.002) effect of time on anterior knee laxity was found from the time of PHV; no changes in estradiol concentration showed up over time (*P* = 0.811). Participants displayed a significant increase (*P* < 0.05) in isokinetic quadriceps strength over time, with no apparent increase in isokinetic hamstring strength.	9 participants were excluded. Females were excluded if they did not meet the developmental inclusion criteria, had a lower limb injury that prevented them from completing the experimental task or had begun menstruating. The hormonal measurements, which were done every 4 months may not have been sensitive enough to detect subtle changes in estrogen during the growth spurt in females.

*PMOS, Pubertal maturational observational scale; DVJ, Drop vertical jump; TS, Tanner (scale) stages; KAM, Knee abduction moment; KAA, Knee abduction angles; GRF, Ground reaction force; PHV, Peak height velocity: peak growth in height during the adolescent growth spurt; BHJMI: Beighton and Horan Joint Mobility Index*.

### Methodological quality assessment

For methodological quality assessment two reviewers (MW and JMS) independently appraised each included study by using Strengthening the Reporting of Observational Studies in Epidemiology (STROBE) (von Elm et al., 2008). STROBE is a checklist of 22 items that relate to the title, abstract, introduction, methods, results, and discussion sections of articles. STROBE is limited to the three (cohort, case-control, cross-sectional) main observational study designs. However, STROBE contributes to a more transparent way of good reporting and generalizability of the research (von Elm et al., 2008). Table 3 shows the percentage from the total number of points of the qualified items for each included study of this review. The average percentage of all included studies is 74%.

**Table 3:** STROBE scores of the quality of reporting of included studies.

**Study**	**Strobe Items out of 22**	**Items %**
Ford et al., 2010	13	59
Hewett et al., 2004	18	82
Hewett et al., 2015	18	82
Quatman et al., 2006	16	73
Quatman et al., 2008	16	73
Steinberg et al., 2006	14	64
Wild et al., 2013	19	86

The Research Triangle Institute Item Bank (RTI-IB) is chosen for evaluating the risk of bias and precision (Table 4; Viswanathan and Berkman, 2012; Viswanathan et al., 2013). It is a practical, transparent, consistent and validated tool used for evaluating risk of bias and precision of observational studies. It captures all of the risk of bias and precision domains (Viswanathan and Berkman, 2012). It is said that the median observed interrater agreement for the RTI-IB was 75% (Viswanathan et al., 2013). For a combination of all items of the RTI-IB it is 93.5% and there is a kappa statistic of 0.88 (Al-Saleh et al., 2012). The RTI-IB covers 11 domains: sample definition and selection, interventions/exposure, outcomes, creation of treatment groups, blinding, soundness of information, follow-up, analysis comparability, analysis outcome, interpretation, and presentation and reporting (Margulis et al., 2014).

**Table 4:**
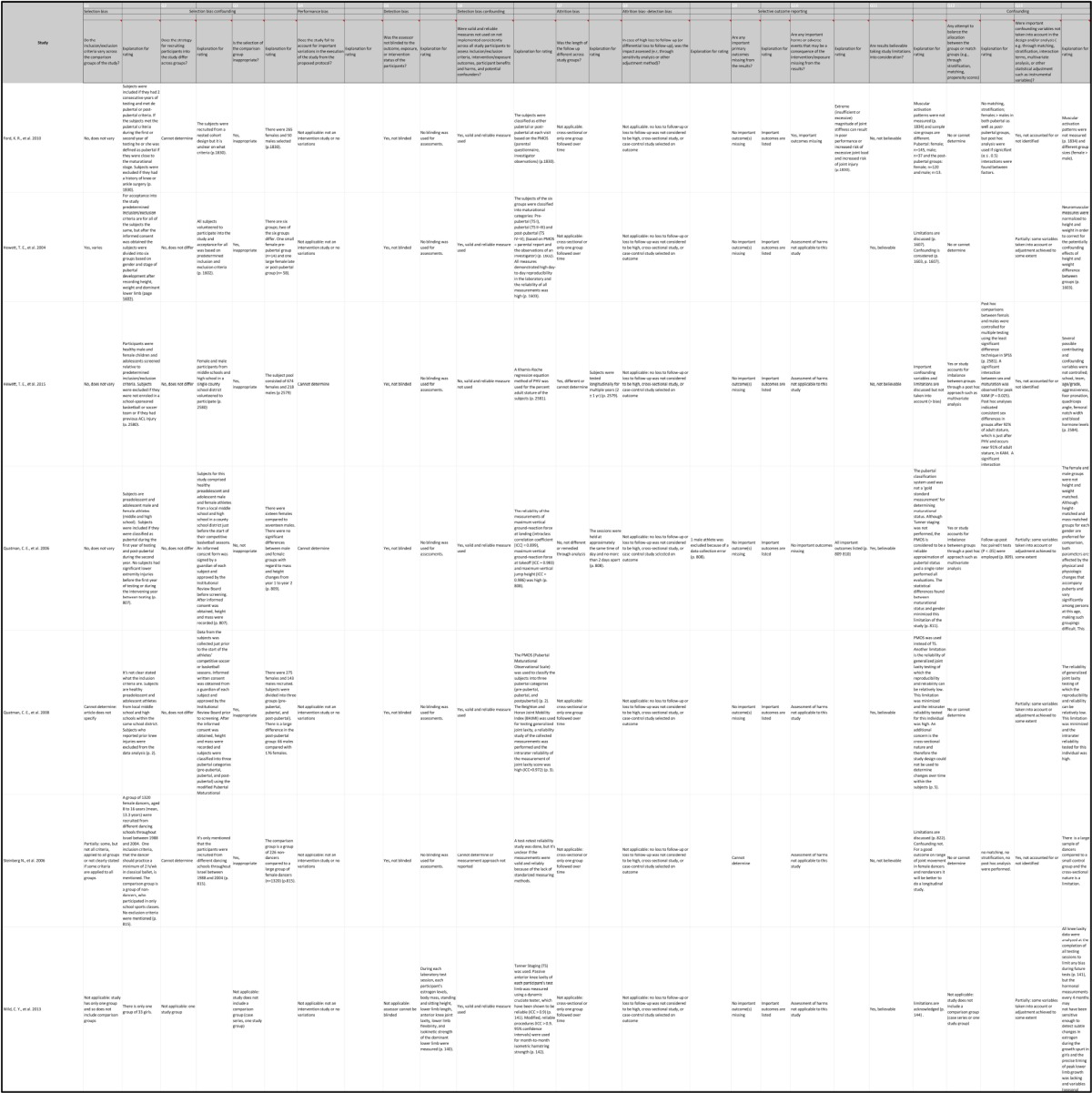
RTI-IB.

*PMOS, Pubertal maturational observational scale; DVJ, Drop vertical jump; TS, Tanner (scale) Stages; KAM, Knee abduction moment; KAA, Knee abduction angles; GRF, Ground reaction force; PHV, Peak height velocity (peak growth in height during the adolescent growth spurt); BHJMI, Beighton and Horan joint mobility index*.

**Figure 2:**
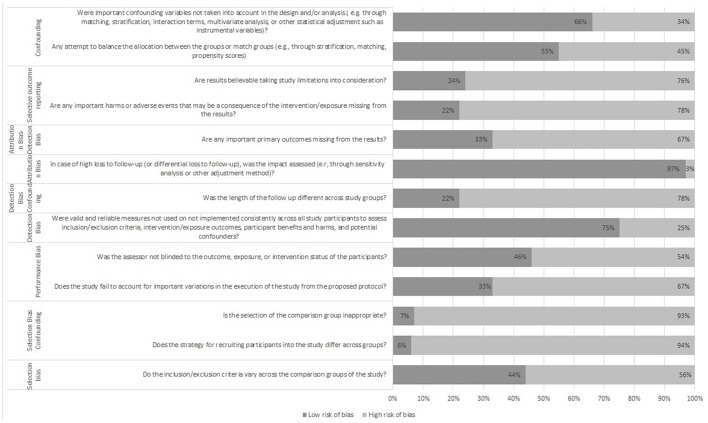
RTI-IB tool.

A black-white figure (Figure 2) is inserted for visual representation of the outcomes of the RTI-IB. It is not easy to summarize all the results of the RTI item bank because there is no good reference to put all of the RTI item bank results together into a summary score (Margulis et al., 2014). Figure 2 shows the risk of bias in all studies (*n* = 7) using the RTI item bank.

## Results

The whole search strategy process and outcome is shown in Figure 1. The search strategy revealed seven articles (Table 2). This present review is based on observational studies: six articles (Hewett et al., 2004, 2015; Quatman et al., 2006; Steinberg et al., 2006; Ford et al., 2010; Wild et al., 2013) have a cross-sectional nature and one study (Quatman et al., 2008) is a cohort. All included studies were published after January 2004 and written in English. The seven studies included a total of 3,418 participants. Four studies (Hewett et al., 2004, 2015; Quatman et al., 2008; Ford et al., 2010) included basketball and soccer players. One study (Steinberg et al., 2006) compared dancers with non-dancers. Another study (Wild et al., 2013) included 33 adolescent females and finally another study (Quatman et al., 2006) included basketball players. For assessing maturation, three studies (Hewett et al., 2004; Quatman et al., 2006; Ford et al., 2010) used the Pubertal Maturational Observational Scale (PMOS), three (Steinberg et al., 2006; Quatman et al., 2008; Wild et al., 2013) used the Tanner Scale and one captured maturation via estimates of percent adult stature (Hewett et al., 2015).

### Participant characteristics

Four studies (Hewett et al., 2004, 2015; Quatman et al., 2008; Ford et al., 2010) included basketball and soccer players. One study (Steinberg et al., 2006) compared dancers with non-dancers. Another study (Wild et al., 2013) included 33 adolescent females and finally another study (Quatman et al., 2006) included only basketball players as participants. The seven studies included a total of 3,418 participants. A detailed overview of the participant characteristics of all the seven included studies can be found in Table 2 of this systematic review.

### Changes in range of motion during the progress through adolescence and growth spurts

All seven included studies (Hewett et al., 2004, 2015; Quatman et al., 2006, 2008; Steinberg et al., 2006; Ford et al., 2010; Wild et al., 2013) showed changes in range of motion during adolescence growth spurts. However, different intrinsic (participants) and extrinsic (e.g., interventions, measurements) factors, (in)dependent variables and covariates needed to be considered when investigating the changes in range of motion during the progress through adolescence and growth spurts.

Four studies (Hewett et al., 2004, 2015; Quatman et al., 2006; Ford et al., 2010) used the Drop Vertical Jump (DVJ) as a dependent variable. These four studies measured different variables during a DVJ. One study (Ford et al., 2010) estimated active joint stiffness of the ankle, knee and hip. The participating adolescence males and females in this study showed an increased active knee stiffness during the DVJ over the span of a year (*P* < 0.05). Males had greater magnitudes of ankle and hip active stiffness (*P* < 0.05) during their growth spurts compared to females. In the post pubertal group, the peak ankle, and hip moments of the males were significantly greater than post-pubertal females (*P* < 0.05). Females had a higher knee to hip moment ratio than males (*P* < 0.05).

Secondly, another study (Hewett et al., 2004) measured the dynamic control of the knee joint by assessing medial knee motion and performed measurements of the lower-extremity valgus angle by assessing knee joint torques. During the progress of maturation, the female athletes had a greater total medial motion of the knees and a bigger maximum lower-extremity valgus angle than did the male athletes during the landing of a jump. Male athletes showed an increased quadriceps peak torque during their process of adolescent growth, whereas girls did not. However, in the prepubertal (*p* < 0.01), early pubertal (*p* < 0.05), and post pubertal (*p* < 0.01) stages hamstrings peak torque was significantly lower in the females than it was in the males. After maturation, the female participants showed more decrease in flexor torques and more differences between the maximum valgus angles of their dominant and non-dominant lower extremities compared with the male participants.

Thirdly, a study (Hewett et al., 2015) determined if there was a relation between skeletal growth and increased knee abduction moment (KAM). This study reported that adolescent females had more increased peak KAM and knee abduction angles (KAA) compared to adolescent males. KAM was at the highest in females at peak height velocity (PHV). After maturation the KAM peak increases in both females and males and after PHV dynamic knee control recovered in male participants and dynamic knee stability is getting less in females. Females showed an increased lower extremity bone length related to greater KAA during maturation, whereas males did not. In conclusion, there seems to be a relationship between increases in peak KAM during and after PHV and increased risk of ACL injury in females.

Finally, the last study (Quatman et al., 2006) of these four studies measured the ground-reaction force (GRF) and vertical jump height (VJH). In this study, male athletes showed increased vertical jump height (VJH) during adolescence growth (*P* < 0.001), whereas female athletes did not. Males significantly had less landing GRF) (*P* = 0.005) compared with females. Take-off force only decreased in females (*P* = 0.003). During maturation, the loading rates decreased in both males and females (*P* < 0.001). Hence, females showed higher loading rates than males (*P* = 0.037) during the progress of adolescence (Quatman et al., 2006).

The three other included studies (Steinberg et al., 2006; Quatman et al., 2008; Wild et al., 2013) focused on different kind of intrinsic factors during the adolescent growth spurt. The first study of these three (Quatman et al., 2008) showed a greater generalized joint laxity in females than in males following the onset of puberty. Females showed increased generalized joint laxity scores between pre-pubertal and post-pubertal groups (*P* = 0.042) as measured by the Beighton and Horan Joint Mobility Index (BHJMI), while males did not (*P* = 0.582). This demonstrates a significant change in laxity during maturation.

Secondly, the other study (Steinberg et al., 2006) showed no changes in range of motion in combined ankle and foot plantar flexion, ankle plantar flexion and hip external rotation in dancers compared with non-dancers. Non-dancers showed a decrease of range of motion with age. Compared to dancers, only ankle dorsiflexion showed a greater ROM in the non-dancer group. The ROM of knee flexion, hip flexion and hip internal rotation decreased with age in both groups. The ROM of hip abduction decreased with age in dancers and stayed the same in non-dancers. Hip extension increased in both groups. The range of motion of the lower back and hamstrings increased with age in dancers and did not change among non-dancers.

Finally, a study (Wild et al., 2013) found a significant effect (*P* = 0.002) of changes on anterior knee laxity over time during adolescent growth. The oestradiol concentration did not change over time (*P* = 0.811). Furthermore, participants showed a significant increase (*P* < 0.05) in isokinetic quadriceps strength over time, but there was no increase in isokinetic hamstring strength over time. This study (Wild et al., 2013) also showed evidence for a significant decrease in hamstring flexibility just before PHV.

### Growth (spurts) and the risk of injuries

During adolescence, the body undergoes major hormonal changes that is accompanied with rapid growth; however, little has been published on how estrogen changes the musculoskeletal system during the growth spurt in girls. One study (Tanner et al., 1976) showed that girls were growing at a rate of 8–10 cm-yr^−1^ during the PHV with greater peak velocity for lower limb growth (4.3 cm-yr^−1^) occurring before the PHV and for torso growth (4–4.5 cm-yr^−1^) after the time of PHV (Tanner et al., 1976). Furthermore, it is mentioned that the growth process within the lower limb is not constant over time (Tanner, 1962). Because of the fast increase in height and the fact that the growth process within the lower limb is not constant over time, girls may show a reduction in lower limb flexibility during their growth spurts, which can contribute to an increased risk of lower limb injuries (Wild et al., 2013).

### Musculoskeletal changes

Males display an increase of velocity in the development of the strength in their quadriceps and hamstrings during PHV and throughout the growth spurt that is not mirrored in females.

Knee flexor and extensor muscles are the most direct active knee joint stabilizers that may protect against an injury during dynamic loading conditions (Ford et al., 2010). Musculoskeletal disbalances between increased quadriceps strength and increased knee laxity at one side and no hamstring strength development at the other side during the adolescent growth spurt in girls, might contribute to a decrease in knee joint stability during landing tasks (Wild et al., 2013) and may contribute to an increase in the risk of anterior cruciate ligament (ACL) injury.

### Decrease in neuromuscular control

There is a difference in neuromuscular patterns between males and females during maturation. Males demonstrate increases in power, strength, and coordination when they are growing older during their maturational stage, whereas females show little change throughout maturation (Hewett et al., 2004; Quatman et al., 2006). During puberty, the increase in vertical jump height and decrease in landing force in male athletes may be caused by a neuromuscular spurt. There is an absence of similar neuromuscular adaptations in female athletes which could be the cause of the reported higher incidence of ACL injuries in females than in males (Hewett et al., 2004). Following the onset of the pubertal growth spurt, the different way of landing from a jump in females may be due to a decreased neuromuscular control of the knee caused by musculoskeletal changes (Hewett et al., 2004). It is due to these musculoskeletal changes and a decrease in neuromuscular control that female athletes showed higher GRFs and higher loading rates at landing compared with males, thereby explaining their higher risk for injury (Quatman et al., 2006).

### Decrease dynamic stability of the knee

The neuromuscular imbalances in adolescent females can contribute to decreased dynamic knee stability (Quatman et al., 2006; Hewett et al., 2015). One study (Quatman et al., 2006) showed that after PHV the tibia and femur length increases, whereas knee flexor strength and recruitment did not. These disbalances during puberty show that growth plays an important role in the mechanism of increased dynamic peak KAM.

This can be a predictor of ACL injury (Hewett et al., 2015).

### Joint stability and joint range of motion

Males and females have the same BHJMI scores before puberty, but it is during puberty that generalized joint laxity increases in female athletes and not male athletes (Quatman et al., 2008). This increase in joint laxity associated with the onset of puberty in females may be related to dynamic instability of the knee, which can lead to a higher risk of ACL injury in female athletes.

The difference in ROM between dancers and non-dancers during their life period of becoming a dancer (ages 8–16 years) depends on different type of joints, movement and age (Steinberg et al., 2006). Steinberg et al. (2006) also showed that hip extension increased in both groups. Hence, dancers' ROM does not improve or decrease with age but rather is preserved.

In short, during the progress through adolescence and growth spurts musculoskeletal changes can lead to; (1) a decrease in knee joint stability during landings from a jump, (2) a decreased neuromuscular control of the knee, (3) the inability to improve neuromuscular control due to pubertal changes, (4) a decrease in dynamic stability of the knee, (5) an increased dynamic peak KAM, (6) an increase in joint laxity and hormonal changes associated with maturation in females. All of these factors may be contributing to changes in ROM and may be related to an increase in the risk of lower extremities. This may explain why the risk of ACL injury is higher in female athletes than in male athletes after puberty.

## Discussion

The present review is the first to investigated whether there are changes in range of motion during the progress through adolescence and accompanying growth spurts in dancers and sportspersons and if these changes can predict the risk of injuries for adolescent dancers and sportspersons.

### Summary of main results

Seven observational studies met the inclusion criteria of this current review and included young adolescent dancers, non-dancers, and sportspersons (Hewett et al., 2004, 2015; Quatman et al., 2006, 2008; Ford et al., 2010; Wild et al., 2013). Young adolescent dancers are at risk for injuries as the result of the inherent demands of dance and the biomechanical changes that occur during growth (Poggini et al., 1999; Caine et al., 2015, 2016). When changes in range of motion during the progress through adolescence and growth spurts are investigated, there need to be considered a lot of variables; intrinsic and extrinsic factors, (in)dependent variables and covariates.

### Range of motion

Unfortunately, there are a few data available on ROM in dancers compared to non-dancers of the same age and relationship between the changes in ROM during the progress through adolescence and growth periods. Steinberg et al. (2006) reported that only ankle dorsiflexion had a greater ROM in the non-dancer group compared to dancers. However, in this study ankle dorsiflexion was only measured in the “parallel” position with extended knees, whereas both demi-plié and grand-plié take place with the hip in full turnout and the knees flexed (Steinberg et al., 2006). It must also be noted that although the authors discussed their study's limitations, they did not include confounding aspects such as the difference in sample size between the two groups, the control group was particularly small compared to the dance group.

### Intrinsic and extrinsic factors

Many authors suggest that intrinsic characteristics, which have a relationship with dance injuries can be measured (Gamboa et al., 2008), though just a few studies have focused on the relationship between measurement of intrinsic characteristics and dance injuries (Gamboa et al., 2008). Research had shown that the prevalence of injury is higher with age and an increase in dance exposure (Bowerman et al., 2015). Extrinsic factors may also have an influence on injury (Coplan, 2002; Negus et al., 2005). It is during the progress through adolescence and growth spurts that students are increasing the intensity of dance training. Therefore, it is difficult to establish if a higher risk of injury is due to age or growth or is a result of more hours of dance training or a combination of all three (Bowerman et al., 2015). The lack of evidence for the increase of ACL injuries may be due to the difficult way of tracking growth and maturation in adolescents, caused by confounding variables (covariates) that are difficult to monitor and control for (Bowerman et al., 2015).

### Injuries

There are several potential risk factors, which places adolescent dancers in the position of suffering a higher risk of overuse injuries, especially lower extremities. These potential risk factors includes growth, the onset of menarche, maturation, menstrual irregularities and poor lower extremity alignment (Bowerman et al., 2015). In addition to different physical demands in ballet, possible explanations for gender differences in injury rates may be hormonal in nature or anatomically based (Caine et al., 2006). Pubertal hormonal changes may have a relationship with increased risk of ACL injury in adolescence females. Estrogen can affect the structure and composition of the human collogen, which can lead to joint laxity (Wild et al., 2013). In this study (Wild et al., 2013) the flexibility of the participants were measured by using joint range of motion tests. However, there were no changes found in hamstring, quadriceps or iliopsoas muscle flexibility noted over time. In this study (Wild et al., 2013) there were hormonal measurements every 4 months, but this may not have been sensitive enough to detect subtle changes in estrogen, lower limb flexibility and potential “at risk” times during the adolescent growth spurt in girls. Also the precise timing of peak lower limb growth was lacking and variables as covariates were not taken into account. Despite all this, it has been speculated that rapid growth, musculoskeletal and hormonal changes might contribute to an increase in knee joint instability during landing tasks. This may cause an increase of ACL injury incidence during the adolescent growth spurt in girls (Wild et al., 2013).

### The risk of injuries

There are other risk factors mentioned for the risk of injuries during the adolescent growth spurt. One study (Ford et al., 2010) investigated if gender had an influence on the relationship between neuromuscular risk factors and knee stiffness during puberty. This study showed that no sex differences were observed in external knee flexor moment and that vertical jump height increased with increased stiffness in males performing the DVJ. It is important to realize that appropriate levels of active joint stiffness, neither insufficient nor excessive, likely results in poor performance or increased risk of excessive joint load and, ultimately, increased risk of joint injury. So, increased stiffness appears to be associated with increased performance. However, this study had different group sizes. There were large pubertal and post pubertal female groups compared with small male groups. Another article (Steinberg et al., 2013) identified the types of injuries for recreational dancers and examined their association with extrinsic and intrinsic factors. This study divided the variables into dependent and independent variables. The conclusion of this study was that joint range of motion and scoliosis can be a potential risk factor for future injury. Secondly, a study also concluded that there is an association between joint range of motion and injuries for many sport disciplines (Gamboa et al., 2008). Another study showed significant associations between hypo and hyper joint range of motion and injuries (Ritter and Moore, 2008).

### Neuromuscular changes

Neuromuscular changes can increase the risk of injuries during the progress through adolescence and growth spurts. It is during this adolescent growth spurt that the tibia and femur grow very fast in both males and females. This can lead to increased torques on the knee joint (Hewett et al., 2004). Due to an increase height and weight the body mass is changing during the onset of puberty. This makes the muscular control of the body more challenging and increases the risk of injury (Hewett et al., 2004). Female athletes showed little neuromuscular changes during the adolescent growth spurt. These little neuromuscular changes may contribute to neuromuscular imbalances and decreased dynamic knee stability (Quatman et al., 2006; Hewett et al., 2015). These disbalances during puberty show that growth plays an important role in the mechanism of increased dynamic peak KAM. This can be a predictor of ACL injury (Hewett et al., 2015). Unfortunately, in this study (Hewett et al., 2004), the number of participants in the female groups differs from the male groups throughout every maturation stage and this study had a cross-sectional nature. Furthermore, two included studies of this present review (Hewett et al., 2004, 2015) had several confounding variables that were not controlled: school, team, age/grade, aggressiveness, foot pronation, quadriceps angle, femoral notch width and blood hormone levels. In short, the high risk of ACL injury in female athletes after maturation may be related to their failure of improving neuromuscular control during puberty (Quatman et al., 2006) as reported in equivalent aged males (Hewett et al., 2004). The positive neuromuscular changes in male athletes manifested themselves in not only being able to generate more power (jump height) but also to control resultant forces, a decrease in ground force reaction on landing (Quatman et al., 2006). For females, the increased landing forces accompanied by a decrease in dynamic knee stability may be a factor in their higher risk of ACL injuries. Despite the differences in neuromuscular patterns between males and females during the progress of adolescence, only a few number of studies have focused on the effects of the differences in neuromuscular patterns and performance between males and females. Unfortunately, most of these reports are cross-sectional in nature and not longitudinal.

### Joint laxity

Before the onset of puberty, males and females have the same BHJMI scores. It is during puberty that generalized joint laxity increases in female athletes and not male athletes (Quatman et al., 2008). This increase in joint laxity may be related to the failure of improving neuromuscular control and an increase of dynamic instability of the knee, which is caused by poor dynamic knee control as a result of the influence of intrinsic factors during puberty (Quatman et al., 2006). However, it need to be considered that the reliability and reproducibility of generalized joint laxity testing used in this study (Quatman et al., 2008) can be relatively low. Hence, this limitation was minimized and the intra-rater reliability tested for this individual was high.

## Limitations

In this current review there are some limitations: there were seven included studies, six of which had a cross-sectional nature and therefore changes over time within the participants could not be determined. Secondly, the generalizability of the findings can be limited for dancers because four of our included studies only focused on soccer and basketball players. One reviewer (JMS) did the search strategy. However, the whole search strategy was done twice by the same author. Another limitation of this present review is that all seven included articles mostly focused on changes in ROM and the risk of injuries in lower extremities, not all joints.

## Conclusion

The results of this review suggest that there are changes in ROM during the progress through adolescence and growth spurts in dancers and sportspersons. These changes may lead to an increase in injury incidence. However, all seven included articles of this present review mostly focused on changes in ROM and the risk of injuries in lower extremities and not all joints. This current review showed that different intrinsic and extrinsic factors, (in)dependent variables and covariates need to be considered when investigating the changes in ROM during the progress through adolescence and the relationship of these changes in ROM with the risk of injury.

In conclusion, there is evidence linking to changes in ROM during the progress through adolescence and the growth spurts in dancers and sportspersons. These changes in ROM may be related to injury incidence. Due to the fact that most included studies in this present review were cross-sectional nature, changes over time within the participants could not be determined. Therefore, further prospective, longitudinal studies are required to investigate the changes in ROM during the progress through adolescence and the relationship of these changes in ROM with the risk of injury.

## New findings

This present review is the first review that investigated whether there are changes in range of motion during the progress through adolescence and growth spurts in dancers and sportspersons and if these changes can predict the risk of injuries for adolescent dancers and sportspersons. This current review showed that there is evidence linking to changes in ROM during the progress through adolescence and growth spurts. These changes in ROM may be related to injury incidence.

## Author contributions

JS: wrote the whole review of abstract, introduction, did search strategy, study selection, data extraction, methodological quality assessment, results, discussion, conclusion, made tables and figures; MW: supervised JS, did study selection, discussed data extraction, did methodological quality assessment, made tables and figures, discussed the whole text of the review, gave comments and advices to JS; RW: discussed parts of the text with JS, gave comments to JS; EB: supervised JS, discussed parts of the text with JS, gave comments and advices to JS.

### Conflict of interest statement

The authors declare that the research was conducted in the absence of any commercial or financial relationships that could be construed as a potential conflict of interest.
