# Supplementary material for: The Relationship Between Range of Motion and Injuries in Adolescent Dancers and Sportspersons: A Systematic Review
Source: Front Psychol. 2018 Mar 22;9:287. doi: 10.3389/fpsyg.2018.00287 (PMC5874564; doi:10.3389/fpsyg.2018.00287)
Supplement: Supplementary file 1 [file DataSheet1.DOCX]

**Appendix 1: Search Strategy**

| **Pubmed (till March 2017)** | range of motion dance adolescence growth spurt, range of motion ballet adolescence growth spurt, range of motion dance adolescent growth spurt, range of motion ballet adolescent growth spurt, range of motion sports adolescent growth spurt, range of motion ballet adolescence growth spurt*, range of motion dance adolescence growth spurt*, range of motion ballet adolescent growth spurt*, range of motion dance adolescent growth spurt*, range of motion sports adolescence growth spurt*, range of motion sports adolescent growth spurt*, range of motion sport adolescence growth spurt*, range of motion sport adolescent growth spurt*, range of motion sport* adolescence growth spurt*, range of motion sport* adolescent growth spurt*, range of motion adolescent growth spurt risk of injury, range of motion adolescence growth spurt risk of injury, range of motion adolescent growth spurt* risk of injury, range of motion adolescence growth spurt* risk of injury, range of motion adolescence growth spurts injury incidence, range of motion adolescent growth spurts injury incidence, range of motion ballet growth spurt* injury incidence, range of motion dance growth spurt* injury incidence, range of motion sports growth spurts injury incidence, range of motion adolescence injuries performance, range of motion adolescence growth spurts injuries performance, range of motion adolescence ballet dance injury performance capabilities, range of motion adolescence ballet dance injury, range of motion adolescence ballet dance injury performance, changes range of motion maturation risk of injury, changes range of motion maturation injury incidence, range of motion adolescence sports risk of injury, range of motion adolescence ballet risk of injury, range of motion adolescence dance risk of injury, range of motion OR ROM risk of injury AND ballet, range of motion OR ROM injury incidence AND ballet, joint range of motion ballet adolescence injury incidence, joint range of motion dance adolescence injury incidence, joint range of motion dance adolescence injury incidence performance, joint range of motion ballet adolescence injury incidence performance, joint range of motion sports adolescence injury incidence performance, joint range of motion sports adolescence risk of injury performance, joint range of motion ballet adolescence risk of injury performance, joint range of motion dance adolescence risk of injury performance, joint range of motion ballet maturation injury incidence, range of motion danc* adolescent growth spurt*,range of motion OR ROM injury incidence AND danc*, range of motion OR ROM danc* risk of injury AND performance, range of motion OR ROM danc* adolescence risk of injury AND performance |
| --- | --- |
| **Cochrane Register of Controlled Trails (CENTRAL): Search (basic) (Title/abstract/keywords) (till March 2017)** | “dancer”, "dancer" AND risk of injury, "dancer" AND "range of motion", "dancer" AND injury incidence, “dancer” AND performance, "dancer" OR range of motion, "dancer" OR injury incidence, range of motion AND dance AND risk of injury, range of motion AND dance AND injury incidence, range of motion AND "sport" AND injury incidence, range of motion AND ballet AND injury incidence, range of motion AND ballet AND risk of injury, range of motion AND "sport" AND risk of injury, "adolescence" AND ballet AND risk of injury, "adolescence" AND dance AND risk of injury, "adolescence" AND "sport" AND risk of injury, "adolescent" AND ballet AND risk of injury, "adolescent" AND dance AND risk of injury, "adolescent" AND "sport" AND risk of injury, "growth spurt" AND ballet AND risk of injury, "growth spurt" AND dance AND risk of injury, "growth spurt" AND "sport" AND risk of injury, "maturation" AND ballet AND risk of injury, "maturation" AND ballet AND injury incidence, "maturation" AND dance AND risk of injury, "maturation" AND dance AND injury incidence, "maturation" AND "sport" AND injury incidence, "maturation" AND "sport" AND risk of injury, growth spurts AND ballet AND performance, growth spurts AND dance AND performance, growth spurts AND “sport” AND performance, "sport" OR ballet OR dance AND adolescence, growth spurts AND ballet, growth spurts AND dance, growth spurts AND sports, growth spurts AND performance, growth spurts AND performance capabilities |
| **Cochrane Database of Systematic Reviews (CDSR): Search manager (Title/abstract/keywords) (till March 2017)** | #1 “dancer”, #2 "dancer" AND risk of injury, #3 "dancer" AND "range of motion", #4 "dancer" AND injury incidence, #5 “dancer” AND performance, #6 "dancer" OR range of motion, #7 "dancer" OR injury incidence, #8 range of motion AND dance AND risk of injury, #9 range of motion AND dance AND injury incidence, #10 range of motion AND "sport" AND injury incidence, #11 range of motion AND ballet AND injury incidence, #12 range of motion AND ballet AND risk of injury, #13 range of motion AND "sport" AND risk of injury, #14 "adolescence" AND ballet AND risk of injury, #15 "adolescence" AND dance AND risk of injury, #16 "adolescence" AND "sport" AND risk of injury, #17 "adolescent" AND ballet AND risk of injury, #18 "adolescent" AND dance AND risk of injury, #19 "adolescent" AND "sport" AND risk of injury, #20 "growth spurt" AND ballet AND risk of injury, #21 "growth spurt" AND dance AND risk of injury, #22 "growth spurt" AND "sport" AND risk of injury,  #23 "maturation" AND ballet AND risk of injury, #24 "maturation" AND ballet AND injury incidence, #25 "maturation" AND dance AND risk of injury, #26 "maturation" AND dance AND injury incidence,  #27 "maturation" AND "sport" AND injury incidence, #28 "maturation" AND "sport" AND risk of injury, #29 growth spurts AND ballet AND performance, #30 growth spurts AND dance AND performance, #31 growth spurts AND “sport” AND performance, #32 "sport" OR ballet OR dance #33 growth spurts AND ballet, #34 growth spurts AND dance, #35 growth spurts AND sports, #36 growth spurts AND performance, #37 growth spurts AND performance capabilities, #38 "sport" or ballet or dance and adolescence |
| **EBSCO Host databases (till March 2017):**  **CINAHL Plus with Full Text, MEDLINE with Full Text, SPORTDiscus with Full Text** | danc*, dancer AND risk of injury, dancer AND range of motion, dancer AND injury incidence, dancer AND performance, adolescence AND ballet AND risk of injury, adolescence AND dance AND risk of injury, adolescence AND sport* AND risk of injury, adolescent AND ballet AND risk of injury, adolescent AND dance AND risk of injury, adolescent AND sport* AND risk of injury, maturation AND ballet AND risk of injury, maturation AND ballet AND injury incidence, maturation AND dance AND risk of injury, maturation AND dance AND injury incidence, maturation AND sport* AND risk of injury maturation AND sport* AND injury incidence, growth spurt* AND ballet, growth spurt* AND dance growth spurt* AND sports, growth spurt* AND performance, growth spurt* AND performance capabilities, growth spurt* AND ballet AND risk of injury, growth spurt* AND dance AND risk of injury, growth spurt* AND sport* AND risk of injury, growth spurt* AND ballet AND performance, growth spurt* AND dance AND performance, growth spurt* AND sport* AND performance, range of motion AND ballet AND injury incidence, range of motion AND ballet AND risk of injury, range of motion AND dance AND injury incidence, range of motion AND dance AND risk of injury, range of motion AND sport* AND injury incidence, range of motion AND sport* AND risk of injury, range of motion AND adolescence AND ballet AND risk of injury, range of motion AND adolescent AND ballet AND risk of injury, range of motion AND adolescence AND dance AND risk of injury, range of motion AND adolescent AND dance AND risk of injury, range of motion AND adolescence AND sports AND risk of injury, range of motion AND adolescent AND sports AND risk of injury, range of motion AND growth spurts AND ballet AND risk of injury, range of motion AND growth spurts AND dance AND risk of injury, range of motion AND growth spurts AND sports AND risk of injury, range of motion AND adolescence AND ballet AND injury incidence, range of motion AND adolescence AND dance AND injury incidence, range of motion AND adolescence AND sports AND injury incidence, range of motion AND adolescence AND ballet AND risk of injury AND performance, range of motion AND adolescence AND dance AND risk of injury AND performance, range of motion AND adolescence AND sports AND risk of injury AND performance, range of motion AND growth spurts AND ballet AND risk of injury AND performance, range of motion AND growth spurts AND dance AND risk of injury AND performance, range of motion AND growth spurts AND sports AND risk of injury AND performance, range of motion AND growth spurts AND ballet AND injury incidence AND performance, range of motion AND growth spurts AND dance AND injury incidence AND performance, range of motion AND growth spurts AND sports AND injury incidence AND performance, range of motion AND growth spurts AND ballet AND injury incidence, range of motion AND growth spurts AND dance AND injury incidence, range of motion AND growth spurts AND sports AND injury incidence, joint range of motion AND ballet AND adolescence AND injury incidence, joint range of motion AND dance AND adolescence AND injury incidence, joint range of motion AND sports AND adolescence AND injury incidence, joint range of motion AND dance AND adolescence AND injury incidence AND performance, joint range of motion AND ballet AND adolescence AND injury incidence AND performance, joint range of motion AND sports AND adolescence AND injury incidence AND performance, joint range of motion AND sports AND adolescence AND risk of injury AND performance, joint range of motion AND ballet AND adolescence AND risk of injury AND performance, joint range of motion AND dance AND adolescence AND risk of injury AND performance |
| **EBSCO Host databases: CINAHL Plus with Full Text, MEDLINE with Full Text, SPORTDiscus with Full Text (till end of March 2017)** | Joint laxity growth spurt dancers, joint mobility growth spurt dancers, changes joint mobility growth spurt dancers, changes range of motion PHV dancers, range of motion PHV young dancers, range of motion PHV young sporters, range of motion PHV sporters, joint flexibility range of motion growth spurt dancers (athletes), range of motion peak height velocity dancers |
| **Embase from 1974 till March 2017** | (range of motion and ballet and risk of injury).ab,kw,ti., (range of motion and dance and risk of injury).ab,kw,ti., (range of motion and sport* and risk of injury).ab,kw,ti., (range of motion and ballet and injury incidence).ab,kw,ti., (range of motion and dance and injury incidence).ab,kw,ti., (range of motion and sport* and injury incidence).ab,kw,ti., (adolescen* and ballet and risk of injury).ab,kw,ti.  (adolescen* and dance and risk of injury).ab,kw,ti., (adolescen* and sport* and risk of injury).ab,kw,ti., (adolescen* and ballet and injury incidence).ab,kw,ti., (adolescen* and dance and injury incidence).ab,kw,ti., (adolescen* and sport* and injury incidence).ab,kw,ti., (growth spurt* and ballet).ab,kw,ti., (growth spurt* and dance).ab,kw,ti., (growth spurt* and sports).ab,kw,ti., (growth spurt* and performance).ab,kw,ti., (growth spurt* and performance capabilities).ab,kw,ti., (growth spurt* AND ballet and risk of injury).ab,kw,ti., (growth spurt* and dance and risk of injury).ab,kw,ti.  (growth spurt* and sport* and risk of injury).ab,kw,ti., (growth spurt* and ballet and performance).ab,kw,ti., (growth spurt* and dance and performance).ab,kw,ti., (growth spurt* and sport* and performance).ab,kw,ti., (range of motion and growth spurts and ballet and risk of injury and performance).ab,kw,ti., (range of motion and growth spurts and dance and risk of injury and performance).ab,kw,ti., (range of motion and growth spurts and sports and risk of injury and performance).ab,kw,ti., (range of motion and growth spurts and ballet and injury incidence and performance).ab,kw,ti., (range of motion and growth spurts and dance and injury incidence and performance).ab,kw,ti., (range of motion and growth spurts and sports and injury incidence and performance).ab,kw,ti., (maturation and ballet and risk of injury).ab,kw,ti., (maturation and ballet and injury incidence).ab,kw,ti., (maturation and dance and risk of injury).ab,kw,ti., (maturation and dance and injury incidence).ab,kw,ti., (maturation and sport* and injury incidence).ab,kw,ti., (maturation and sport* and risk of injury).ab,kw,ti |
| **Journal of Dance, Medicine and Science (JDMS)** | Manual search of the Journal of Dance, Medicine and Science (from March 1997 till March 2017) was done and resulted in 14 publications (title/abstract). |
